# Modern Sociology

**Published:** 1900-09-01

**Authors:** 


### Modern Sociology.

### FIGHTING A FAMINE.

While everyone is more or less interested in the work being done by the representatives of English power in India in relieving famine, very few have any clear idea what the work of famine relief is, how it is done, and what is done in advance to prevent famine assuming serious proportions. In the old days no doubt it was thought that famines came by the will of God, and had to be submitted to accordingly. Therefore, the Oriental starved meekly to death, and cursed nobody. Nowadays he looks to the British Government for help, and feels wronged when, even if life is preserved, some degree of comfort has to be sacrificed. Perhaps in some ways famines are more severe now than they were in the days of old, because the population is denser. Not that India is densely populated, from an English standpoint. In this country there are about 500 persons to the square mile, and in India only 185; but India feeds its own population, while Britain buys the food for its millions with the products of its manufactures. A self-supporting, self-feeding nation means a nation largely occupied in agriculture, and this is what we find in India. This means also that a larger proportion of the population will suffer at once and directly by any failure of the crops. Since the *Pax Britannica* has prevented the periodic decimation of the people by war they have increased in number, and consequently some have had to till the less productive lands that were formerly left waste. On these lands only a bare subsistence is obtained; the crop of one year serves to keep the cultivator and his family in food till the next. On these people the failure of the rainfall acts at once; they must be relieved or starve, while the fortunate possessors of fertile land which bears a harvest sufficient for the needs of two or more years can not only live but even gain some wealth, when grain, being scarce, reaches a higher price. The village artisans suffer almost as much as the poorer cultivators, for they are paid in kind for their work, and when their employers starve they starve with them.

The development of the science of meteorology has done much to lessen the evils of famine in India. It is now known that in certain districts a periodical failure of rainfall is to be expected every 12 years. The districts vary in different years, and the sum of present observations indicates that some part of the country will be visited by famine every four years. It might seem that in such a case "forewarned is forearmed," and to a great extent—much greater than people at home imagine—this is the case. But the caste system, the iron rule of prejudice, and the traditional habits of the people, make it much more difficult to feed the starving inhabitants of, say, the Deccan, with the superfluity of the Punjaub, than it is to feed London from Argentina or Minnesota. Wheat can be bought in any of the continents, but wheat is the staple food of the natives of a part of the north-west only. There is plenty of rice in Burmah, but, except in some well-watered districts where famine rarely comes, rice is eaten only by the upper classes. The bulk of those whom famine is most likely to affect live habitually on millet, a seed not much cultivated in other countries. And there is great difficulty in persuading the Indian peasant to change his habitual food. This is the characteristic of the uneducated all the world over. There has been an attempt made to popularise maize as a food, which would be a great boon, seeing that it can be imported at half the price of wheat; but old prejudice is hard to fight against. Still it is being fought; and the great development of the railway system in India is helping in the fight, both by lessening that isolation of communities which tends to narrowness of view, and by bringing the abundance of the prosperous districts to supply the needs of the poverty-stricken.

In this sense the railways may fitly be regarded as relief works. They are so in another fashion, since work on them is one of the ways by which the Government helps the sufferers from famine. It is better to give work than to give alms, and, above all, it is better to give work which will tend to prevent famine being so severely felt when next it visits the district. The making of canals, and other methods of irrigation are also useful in this double way. The canal is perhaps over-rated by European minds as a remedial agent. For one thing, there are some sandy soils which absorb the water almost as fast as it is poured in. For another, if the water of the canal is derived from a stream within the famine-stricken district the supply is likely to fall short just when the lack of rain renders it most desirable as an irrigating agent. Of more general utility are the irrigation wells, of which about 20,000 have been sunk in the Madras Presidency, and a smaller number in more recent years in Upper India. These irrigate from four to eight acres each, and the State is ready to lend money for the making of them on the security of the land to be irrigated.

Long before a famine breaks out in any particular district, the State has made inquiries as to the probable needs that may arise and the measures which, in the opinion of local observers, will best tend to lessen suffering. Public works, large or small, are prepared for, and the district is mapped out into divisions, over each of which an inspector will be placed as soon as the famine appears. In administration the Famine Code deals with every conceivable detail, and requires only to be acted upon with intelligence. The pay given on relief works is, of course, very small. Intended as they are only to meet an emergency, it is undesirable to pay such wages as will withdraw such cultivators as can live at all from their regular work. To lessen the area under cultivation by offering inducements to the cultivating class to take up other employments would, as India stands to-day, be to make famine permanent. The relief works provide for the able-bodied of all ages and either sex; for Indian women and children are accustomed to share the family tasks, and going to works at a distance does not entail the breaking up of family ties. The infirm are cared for in poorhouses in the various villages. In these poorhouses one or two natives of high caste are employed to prepare food, for a Hindoo is degraded for ever if he accepts food at the hand of one of lower caste than himself. It is such prejudices as these that make Indian administration so full of difficulty.

